# The impact of antiandrogen 2-hydroxyflutamide on the expression of steroidogenic enzymes in cultured porcine ovarian follicles

**DOI:** 10.1007/s11033-014-3291-6

**Published:** 2014-03-02

**Authors:** Malgorzata Duda, Malgorzata Grzesiak, Malgorzata Knet, Katarzyna Knapczyk-Stwora, Zbigniew Tabarowski, Agata Michna, Maria Slomczynska

**Affiliations:** 1 Department of Endocrinology, Institute of Zoology, Jagiellonian University, Gronostajowa 9, 30-387 Cracow, Poland; 2 Department of Experimental Hematology, Institute of Zoology, Jagiellonian University, Gronostajowa 9, 30-387 Cracow, Poland

**Keywords:** 2-Hydroxyflutamide, Steroidogenic enzymes, Ovarian follicles, Pig

## Abstract

We used our model system for agonism and antagonism of the androgen receptor (AR), in which the porcine ovarian follicles were exposed on the excessive concentration of an AR agonist- testosterone (T) or an AR antagonist- 2-hydroxyflutamide (2-Hf) to: (1) analyze the spatiotemporal expression of ovarian 3β-hydroxysteroid dehydrogenase (3β-HSD), cytochrome P450 17α-hydroxylase/c17,20-lyase (P450c17) and cytochrome P450 aromatase (P450arom); (2) to determine the contribution of AR-mediated action during steroidogenesis and (3) to establish some correlations between the onset and expression pattern of the investigated proteins. Whole follicles (6–8 mm in diameter) isolated from mature porcine ovaries have been incubated (for 24 h) in an organ culture system in the presence of T (10^−7 ^M), 2-Hf (1.7 × 10^−4^ M) or both T and 2-hydroxyflutamide (T+2-Hf, at the same concentrations as when added separately). Thereafter, sections obtained from cultured follicles were processed for main steroidogenic enzymes detection by immunohistochemistry. Moreover, expression of their mRNA and protein was determined by real-time PCR and Western blot analysis. Progesterone, androgens and estradiol concentrations in the culture media were measured by radioimmunoassays (RIA). Our results demonstrated that 2-Hf can influence the steroidogenic activity of porcine follicles in vitro through the blockade of AR. It was shown that follicular 2-Hf treatment brought about dramatic decline in the production of the investigated steroids. What is more the addition of 2-Hf separately caused a negative effect on 3β-HSD and P450c17 mRNA and protein expression by ovarian follicles, while it was without effect on P450arom mRNA level. Quite opposite effect was observed in case of the simultaneous addition of 2-Hf and T. It caused high increase, in both P450arom mRNA and its protein. What was interesting, addition T+2-Hf evoked 3β-HSD and P450c17 increase on mRNA level, but decreased their protein expression. This was against our expectations but the reason for that finding remains undiscovered, intriguing and worth reporting. These results suggest that alike, steroidogenic enzymes activity and their expression is associated with the presence of androgens and AR in the porcine ovary.

## Introduction

Female reproduction depends on a succession of multiple integrated processes in which the ovary has two important roles: during follicular development produces eggs for fertilization and is an important site of steroids production whose concentrations are tightly regulated by feedback control mechanisms [1–3]. Androgens are one of the most important agents influencing folliculogenesis. Their biological action is primarily exerted through transcriptional regulation by the nuclear androgen receptor (AR), but the molecular cascades governed by AR remain largely unknown. Androgens can modulate follicular function by interactions with various factors to promote granulosa cells (GC) differentiation [4]. On the other hand, they can antagonize follicular development by inducing apoptosis in GC and in this way can promote follicular atresia [5]. Porcine GC are considered to be the main site of AR-mediated androgen action in the follicle because of its strongest expression there, as it was stated in both the whole follicles [6] and GC in vitro [7]. The presence of AR within GC provides basis for the interactions with AR agonists and antagonists. The agonist- or antagonist- bound steroid hormone receptors regulate gene expression by binding to the regulatory elements in promoters of susceptible genes, in a tissue-dependent manner. At present, there is an imminent concern that environmental man-made chemicals endocrine disrupting chemicals (EDCs) with antiandrogenic properties [8], among others, are capable of modulating hormonal responses, thereby interfering with normal physiological processes that are critical to fertility. In this work we focused on 2-hydroxyflutamide (2-Hf) known to interact with the AR. 2-Hf is the biologically active form of flutamide (FLU), which is the first androgen-receptor blocker to achieve a wide-spread use [9, 10]. FLU was used to treat prostate cancer, where it competes with testosterone (T) for binding to AR, thereby reducing the growth of cancer cells [11]. It reduced fecundity in the fathead minnow by decreasing oocytes maturation in females and causing spermatocyte degeneration and necrosis in the testis [12]. The antiandrogenic activity of 2-Hf and FLU has been well established [13–16], what is interesting it has been demonstrated that 2-Hf is approximately 1–5 times more potent than its parental compound, FLU [17].

Steroidogenic enzymes are responsible for the biosynthesis of various steroid hormones including progestins, androgens and estrogens from cholesterol. Among them there are several specific cytochrome P450 enzymes (CYPs), hydroxysteroid dehydrogenases (HSDs) and steroid reductases [18]. De novo synthesis of all steroid hormones starts with the conversion of cholesterol to pregnenolone by cholesterol side-chain cleavage (CYP11A1) [19]. Pregnenolone is then converted to progesterone by 3β- hydroxysteroid dehydrogenase (3β-HSD), which is found both in the mitochondria and smooth endoplasmic reticulum. Pregnenolone and progesterone are the precursors for all other steroid hormones among others androgens. Cytochrome P450 17α-hydroxylase/c17,20-lyase (P450c17) is the next important enzyme in the steroidogenic pathway leading to androgen synthesis [20, 21]. It is a member of cytochrome P450 group of oxydases [18], which is a single protein possessing both hydroxylase and lyase activities and catalyzes the conversion of pregnenolone and progesterone to their corresponding androgen products, dehydroepiandrostenedione and androstenedione, respectively [21]. The ovum inside the developing follicle is directly surrounded by layers of GC followed by theca cells (TC), which is where the steroidogenesis predominantly takes place. The theca interna is highly vascularized and produces large amounts of progesterone and androgens. Androstenedione and testosterone diffuse into the neighboring poorly vascularized GC where they are converted predominantly to estradiol via concerted action of another important steroidogenic enzyme, aromatase (P450arom).

Antiandrogens administered both as medical treatment and present in the environment belong to EDCs since they have abilities not only to interact directly to activate or antagonize sex hormone receptors, such as AR [22] but also enzymes involved in the steroid biosynthesis pathway are being recognized as important targets of their action. Interferences with steroid biosynthesis may result in an impaired reproduction and alterations in sexual differentiation, growth and development. Particularly 3β-HSD, P450c17 and P450arom have been the subject of studies into the mechanisms by which antiandrogens interfere with sex steroid hormone homeostasis and function [18]. Therefore it is possible for antiandrogens to cause or contribute to the hormonal disruption and subsequent reproductive and developmental toxicities by interfering with the function of those key enzymes involved in steroids synthesis and breakdown. Results reported by Ayub and Level [23] demonstrated that antiandrogens may have an inhibitory effect on androgen biosynthesis which could prove to be of clinical significance. It was shown that 2-Hf is a competitive inhibitor of P450c17 activity in rat testes. Unfortunately still relatively little is known about underlying mechanisms of the interference of 2-Hf with steroidogenesis and potential impact on the ovary. On the basis of these findings the aim of the present experiments was to elucidate the mechanism of action of 2-Hf at the follicular level by examining whether 2-Hf affects steroidogenesis. What is more, it was attempted to determine, by the examination of main steroidogenic enzymes immunolocalization, the contribution of AR-mediated actions during steroidogenesis and establish some possible correlations between the onset and expression pattern of the investigated proteins.

## Materials and methods

### Sample collection

Porcine ovaries were obtained from Polish Landrace gilts at a local slaughterhouse and placed in a cold phosphate-buffered saline (PBS; pH 7.4, PAA The Cell Culture Company, Dartmouth, MA, USA) containing Antibiotic/Antimycotic Solution (AAS 10 μl/ml; PAA The Cell Culture Company). Ovaries were transported to the laboratory within 30 min and rinsed twice with sterile PBS supplemented with antibiotics. In each experiment, ten ovaries from five animals were selected for isolation of follicles. Assuming that each ovary yielded 3–5 follicles, the total number of follicles varied from 30 to 50. The phase of the estrous cycle was determined according to the established morphological criteria [24]. Medium follicles (6–8 mm in diameter), classified by morphometric criteria as healthy ones [25], were selected for organ cultures. This procedure has been chosen to minimize the variability between tissues and animals.

### Follicle culture and preparation for immunohistochemical analysis

Whole follicles (*n* = 36, 6–8 mm in diameter) isolated from porcine ovaries were cultured on a filter disk on a triangular stainless steel grid over a well of McCoy’s 5A medium supplemented with 10 % FBS and AAS (5 μ/ml) [26].

Experimental cultures were carried out in the presence of T (10^−7 ^M) and 2-Hf (1.7 × 10^−4 ^M) separately or in combination, T and 2-Hf (T+2-Hf). All culture media—control and experimental ones- were collected after 24 h and stored at −20 °C for further progesterone, androgens and estradiol level analysis, whereas follicles (*n* = 3/each group) were fixed in 4 % paraformaldehyde, subsequently dehydrated in an increasing gradient of ethanol and embedded in paraplast (Sigma-Aldrich, St. Louis, MO, USA). 5 μm thick sections were mounted on slides coated with 3-aminopropyltriethoxysilane (Sigma-Aldrich), deparaffinized, and rehydrated through a series of decreasing alcoholic solutions.

### Immunohistochemistry

Sections of follicles were subjected to a microwave oven 3 × for 4 min, in 0.01 M citric acid buffer (pH 6.0), to retrieve antigens. Endogenous peroxidase activity was blocked by incubation with 0.3 % H_2_O_2_ in TBS (Tris-buffered saline, pH 7.4), and non-specific binding was blocked for 30 min by incubation with 5 % normal horse serum (NHS, Sigma-Aldrich) for P450arom or 5 % normal goat serum (NGS, Sigma-Aldrich) for P450c17 and 3β-HSD. Then, sections were incubated overnight at 4 °C with either a monoclonal mouse anti-human cytochrome P450 arom antibody (AbD Serotec, UK), at a dilution 1:50, or with a polyclonal anti-recombinant mouse 3β-HSD (a gift from Professor Anita H. Payne, Stanford University Medical Center, CA, USA), at a dilution 1:1,000, or with a polyclonal rabbit anti-porcine P450c17 (a gift from Professor Dale B. Hales from Southern Illinois University, Carbondale, USA), at a dilution 1:100. Control sections were incubated with 5 % NHS or NGS instead of the primary antibody. The antigens were visualized using biotynylated secondary antibodies—horse anti-mouse antibody (for monoclonal primary antibody) or goat anti-rabbit antibody (for polyclonal primary antibodies) (1:300, 1.5 h at RT; Vector Laboratories), avidin-biotin-peroxidase complex (1:100, 40 min at RT; StreptABComplex-HRP, Vector Laboratories), and 3, 3′-diaminobenzidine as the substrate. Slides were dehydrated and mounted in DPX (Sigma-Aldrich). The intensity of immunoreaction was analyzed on each section.

### Quantitative evaluation of staining intensity

The sections were photographed using the Nikon Eclipse E200 microscope attached to the Coolpix 5400 digital camera (Nikon, Tokyo, Japan) with corresponding software. To estimate the intensity of immunoreaction quantitatively, image processing and analyses were performed on six different sections from each examined follicle and were analyzed using the public domain ImageJ software (National Institutes of Health, Bethesda, MD, USA). The source images were converted to 8-bit grayscale images and the intensity of staining was measured detachedly using point selection tool. Moreover, the background was analyzed as well. Results from each sample were saved as individual mean and interpolated to the following formula:

${\text{ROD}} = {\text{OD}}_{\text{specimen}} /{\text{OD}}_{\text{background}} = { \log }\left( {{\text{GL}}_{\text{blank}} /{\text{GL}}_{\text{specimen}} } \right)/{ \log }\left( {{\text{GL}}_{\text{blank}} /{\text{GL}}_{\text{background}} } \right)$where *GL*—grey level for stained area (specimen) and unstained area (background) and blank as a grey level measured after removing the slide from the light path. The intensity of immunoreaction was expressed as relative optical density (ROD).

### Western blot analysis

Cultured porcine follicles were homogenized on ice with a cold Tris/EDTA buffer (50 mM Tris, 1 mM EDTA, pH 7.5), sonicated and centrifuged at 10,000×*g* for 20 min at 4 °C. Supernatant was collected and stored at −20 °C. Protein concentration was determined with Bradford reagent (Bio-Rad Protein Assay; Bio-Rad Laboratories GmbH, München, Germany) using bovine serum albumin as a standard. Aliquots of follicle homogenates containing 20 μg of protein were solubilized in a sample buffer consisting of 62.5 mM Tris–HCl pH 6.8, 2 % SDS, 25 % glycerol, 0.01 % bromophenol blue, 5 % β-mercaptoethanol (Bio-Rad Laboratories) and heated for 3 min at 99.9 °C. After denaturation, samples were separated via 12 % SDS–polyacrylamide gel electrophoresis under reducing conditions according to Laemmli [27]. Separated proteins were transferred onto a nitrocellulose membrane using a wet blotter in the Genie Transfer Buffer (20 mM Tris, 150 mM glycine in 20 % methanol, pH 8.4) for 90 min at a constant voltage of 135 V. After overnight blocking with 5 % non-fat milk in TBS, 0.1 % Tween 20 (dilution buffer) at 4 °C with gentle shaking, the membranes were treated with the primary antibody (for details see immunohistochemistry subchapter: anti P450 arom, dilution 1:1,000; anti P450c17, dilution 1:1,000; anti 3β-HSD, dilution 1:10,000) for 1.5 h at room temperature (RT). The membranes were washed and incubated with a secondary antibody conjugated with the horseradish-peroxidase labeled goat anti-rabbit IgG (for polyclonal primary antibodies) or horseradish-peroxidase labeled horse anti-mouse IgG (for monoclonal primary antibody) (Vector Laboratories; dilution 1:3,000) for 1 h at RT. The signals were detected by chemiluminescence using Western blotting Luminol Reagent (Santa Cruz Biotechnology). The blots were visualised using the ChemiDoc™ and all of the bands were quantified using the Image Lab™ 4.0 Software (BioRad Laboratories).

### RNA isolation and cDNA preparation

Total cellular RNA from incubated ovarian follicles, were isolated using Tri Reagent solution (Ambion, Austin, TX, USA) following the manufacturer’s instruction. RNA quality was checked on a 1 % formaldehyde-agarose gels and the concentration was quantified using NanoDrop ND2000 Spectrophotometer (Thermo Scientific, Wilmington, DE). 1.5 μg of total RNA was reverse transcribed using a High-Capacity cDNA Reverse Transcription kit (Applied Biosystems, Carlsbad, CA, USA) according to the manufacturer’s protocol. Reverse transcriptase reaction mixtures were prepared in 20 μl volume using the random primers, dNTP mix, RNAse Inhibitor and Multi Scribe Reverse Transcriptase. Genomic DNA amplification contamination was checked periodically by control experiments, in which reverse transcriptase was omitted during the RT step. The reverse transcription was performed in a Veriti Thermal Cycler (Applied Biosystems) with a temperature cycling program of 10 min at 25 °C, 2 h at 37 °C, and 5 min at 85 °C, with subsequent cooling to 4 °C. Samples were kept at −20 °C until further analysis.

### Real-time PCR quantification and data analysis

Real time PCR was performed using the StepOne™ Real-Time PCR System (Applied Biosystems). The mRNA expression level of the HSD3B1, CYP17A1 and CYP19A1 were quantified in each sample using TaqMan Gene Expression Master mix (Applied Biosystems) and porcine-specific TaqMan Gene Expression assays (Applied Biosystems) for HSD3B1 (assay ID: Ss03391751_m1), CYP17A1 (assay ID: Ss03394947*_*m1) and CYP19A1 (assay ID: Ss03384876_u1) with endogenous control for glyceraldehyde-3-phosphate dehydrogenase (GAPDH; assay ID: Ss03375629_u1) according to the manufacturers’ instructions. Quantitative PCR was performed with 200 ng of cDNA, 1 μl TaqMan Gene Expression Assay, and 10 μl TaqMan PCR master mix in a final volume of 20 μl. The reactions were incubated in a 96-well optical plate at 95 °C for 10 min, followed by 40 cycles of 95 °C for 15 s and 60° for 1 min to determine the cycle threshold (Ct) number for quantitative measurement.

Relative quantification (RQ) was obtained using the 2^−ΔΔCt^ method, adjusting the HSD3B1, CYP17A1 and CYP19A1 mRNA expression to the expression of GAPDH mRNA and considering the adjusted expression in the control samples as reference (RQ = 1) [28]. Data were expressed as mean RQ ± standard error of the mean (SEM).

### Radioimmunoassay

Steroid concentrations were determined in culture media of ovaries using specific radioimmunoassay as described previously by Szoltys et al. [29]. Progesterone was measured using [1,2,6,7- 3H]progesterone (specific activity 96 Ci/mmol; GE Healthcare, Amersham International, Little Chalfont, Bucks., UK) as a tracer and an antibody induced in sheep against 11α-hydroxyprogesterone succinyl: BSA (a gift from Professor Brian Cook, University of Glasgow, Glasgow, Scotland). The lower limit of sensitivity of the assays, was in the order of 20 pg. Coefficients of variation, within and between assays, were below 5.0 and 9.8 %, respectively.

Androgens were measured using [1,2,6,7-3H]testosterone (specific activity 81 Ci/mmol; GE Healthcare, Amersham Int.) as a tracer and rabbit antibody against testosterone-3-0- MO:BSA (a gift from dr Bela Ričařova, Institute of Radiology, Czech Academy of Sciences, Prague, Czech Republic). The lower limit of sensitivity was in the order of 5 pg. Cross-reaction of this antibody was 18.3 % with dihydrotestosterone, 0.1 % with androstendione and less than 0.1 % with other major ovarian steroids. Since this antibody also significantly bound dihydrotestosterone, the measured steroids were referred to as androgens rather than testosterone. Coefficients of variation within and between assays were below 5.0 and 9.7 %, respectively.

Estradiol-17β was determined using [2,4,6,7-3H] estradiol (specific activity 104 Ci/mmol: GE Healthcare, Amersham Int.) as a tracer and rabbit antibody against estradiol-17-Ocarboxymethyloxime: BSA (a gift from Professor Roman Rembiesa, Institute of Pharmacology, Polish Academy of Sciences, Krakow, Poland). The lower limit of sensitivity of the assays was 5 pg. Cross-reaction was 1 % with keto-estradiol-17β, 0.8 % with estrone, 0.8 % with estriol, 0.01 % with testosterone and less than 0.1 % with major ovarian steroids. Coefficients of variation within and between assays were below 4 and 7.5 %, respectively.

For and androgen assays, appropriate aliquots of culture media were extracted with 2.5 ml ethyl ether. All samples were assayed in duplicate.

### Statistical analysis

Statistical analysis was performed using Statistica 5.1 software (StatSoft Inc., Tulsa, OK, USA). The number of separate experiments for whole follicles incubations was *n* = 3 (follicles was performed in triplicate). Shapiro–Wilk W test was used for normality. The significance of differences between control and experimental incubations for immunohistochemical data, Western- blot data and quantitative RT-PCR data were assessed using the non-parametric Mann–Whitney’s test, whereas radioimmunological data were assessed using Student’s *t* test. Western blot and quantative RT-PCR analysis was performed for each experiment and repeated three times. The data were presented as mean ± SEM and were statistically evaluated with significance at *p < 0.05, **p < 0.01, ***p < 0.001.

## Results

### Steroidogenic enzymes immunolocalization

**Fig. 1:**
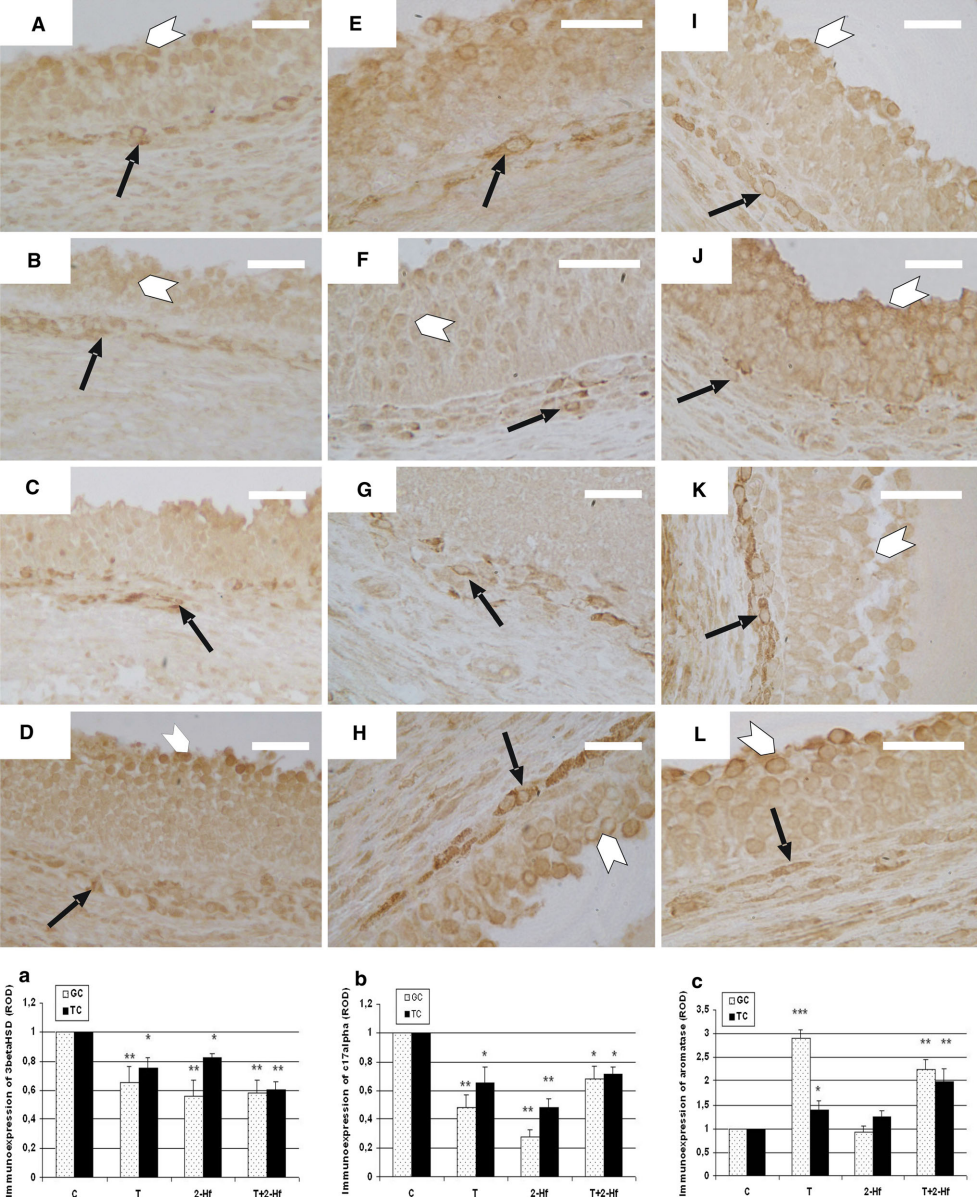
The effect of 24 h antiandrogen treatment on the immunostaining for 3β-HSD (**a**–**d**), P450c17 (**e**–**h**) and P450arom (**i**–**l**) after whole porcine follicles incubations. Control cultures (**a**, **e**, **i**), cultures under T influence (**b**, **f**, **j**), cultures under 2-Hf influence (**c**, **g**, **k**), cultures under T+2-Hf influence (**d**, **h**, **l**). Cytoplasmic staining of steroidogenic enzymes in granulosa cells (*white arrowheads*), cytoplasmic staining in theca cells (*black arrows*). Quantitative analysis of the intensity of steroidogenic enzymes staining expressed as relative optical density (ROD) of diaminobenzidine brown reaction products in follicle cultures (**a**, **b**, **c**) exposed to an androgen agonist (T), an antagonist (2-Hf) or both (T+2-Hf) versus respective controls. Values are mean ± SEM. *Asterisks* indicate significant differences between control and experimental cultures. Significant differences from control values are denoted as *p < 0.05, **p < 0.01, Mann–Whitney’s test. Bar = 50 μm (**a**–**d**, **g**–**j**), bar = 20 μm (**e**, **f**, **k**, **l**)

In all carried out variants of follicular cultures, the granulosa layer displayed a gradient of cytoplasmic 3β-HSD immunoreaction intensity (Fig. 1a–d) with the strongest immunolabelling in the antral granulosa cells, and a much weaker one in the cells lying in closer proximity to the theca interna layer that is mural granulosa cells. A weakly positive reaction was found in granulosa cells of follicles cultured under the 2-Hf influence. These cells showed an almost negative 3β-HSD labeling with single cells demonstrating a strong immunoreaction (Fig. 1c). The most intense 3β-HSD immunolabeling was observed in the theca interna cells of control follicles (Fig. 1a) in contrast to the follicles incubated with the addition of T+2-Hf (Fig. 1d) which demonstrated a quite weak 3β-HSD immunolabeling.

In granulosa layer of control follicles and those cultured with the addition of T+2-Hf, the strongest P450c17 immunolabeling was limited to the antral granulosa cells with a very strong immunoreaction found in peripherally located cells (Fig. 1e, h). A weak and evenly distributed P450c17 immunolabeling was found in granulosa cells of follicles incubated with the addition of 2-Hf to the culture medium (Fig. 1g). The strongest P450c17 immunoreaction was localized in theca interna cells of control follicles (Fig. 1e). Theca interna cells of T+2-Hf treated follicles also exhibited a distinct cytoplasmic reaction (Fig. 1h).

Immunostaining for P450arom was observed in various cell types of the follicles, both control and experimental ones (Fig. 1i–l). In follicles cultured under the influence of T and T+2-Hf, the intensity of immunostaining in GC was the strongest (Fig. 1j), whereas the number of positively stained cells was dramatically lower in follicles cultured with the addition of 2-Hf. What is more, a decrease in the number of stained GC was accompanied with an increase in especially strong P450arom immunoreaction observed in theca interna cells (Fig. 1k). The omission of primary antibodies resulted in a lack of 3β-HSD, cytochrome P450c17, and cytochrome P450arom labelling in control sections (not illustrated). Quantitative evaluation of the intensity of the immunohistochemical reaction in the whole follicles expressed as relative optical density (ROD) generally confirmed the qualitative data (Fig. 1a–c).

### Western blot analysis

**Fig. 2:**
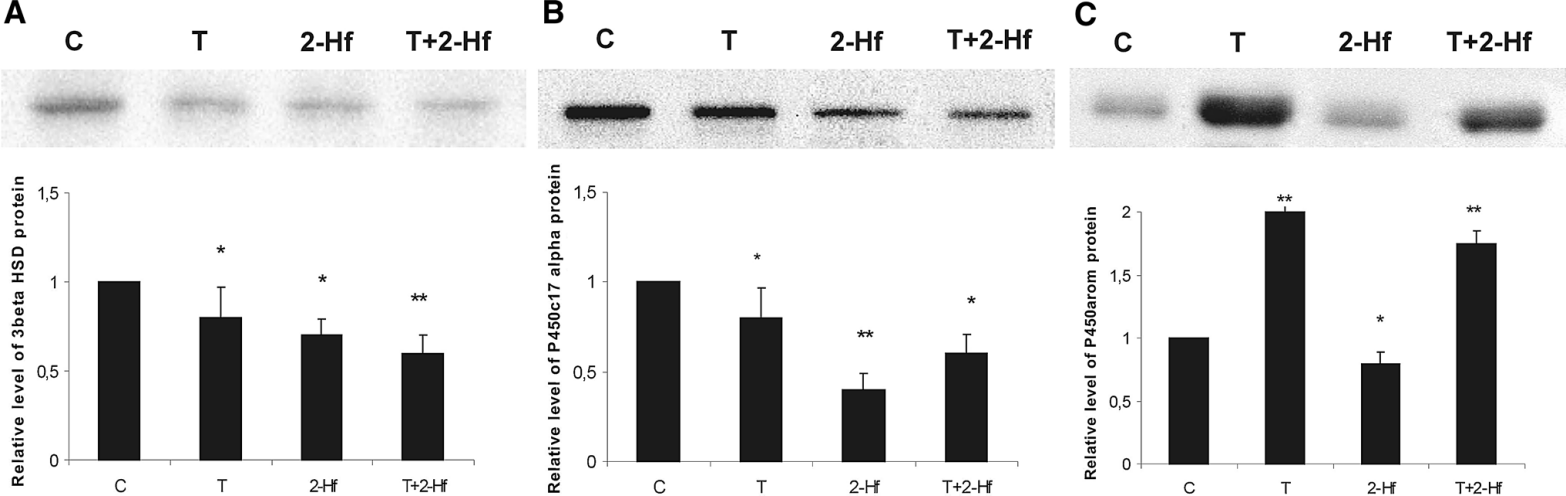
Representative Western blot analysis of 3β-HSD **a**, P450c17 **b** and P450arom **c** protein expression levels in homogenates of control, testosterone (T), 2-hydroxyflutamide (2-Hf), testosterone and 2-hydroxyflutamide (T+2-Hf) treated follicles after 24 h cultures. Data obtained from three separate analyses are expressed as mean ± SEM (*n* = 3). Significant differences from control values are denoted as *p < 0.05 and **p < 0.01, Mann–Whitney’s test

To provide further evidence that whole follicles express 3β-HSD, P450c17 and P450arom protein and to confirm the specificity of the antibodies used, Western blot analysis was performed. Using the anti-mouse 3β-HSD, anti-porcine P450c17 and anti-human cytochrome P450 aromatase antibodies a single bands of 42, 55 and 55 kDa, respectively were detected in all investigated probes, either control ones or exposed to the androgen agonist or antagonist or both. Immunoblotting was also used for β-actin, which served as a control for equal protein loading. The bands representing each data point were densitometrically scanned, and the data obtained for the investigated proteins were normalized against β-actin. The protein level within the control group was arbitrarily set as 1, against which statistical significance was analyzed. In case of 3β-HSD all applied agents generated a significant decrease in protein level (*p* < 0.05 or *p* < 0.01, Fig. 2a). Quantitative analysis of relative protein levels revealed a distinct decrease in P450c17 level in whole follicles exposed to both 2-Hf and T+2-Hf (*p* < 0.01 and *p* < 0.05, respectively; Fig. 2b). In case of P450arom level quite opposite effects were observed. The addition of T or T+2-Hf caused an increase in this protein level (*p* < 0.01). 2-Hf addition brought about significant decrease in P450arom protein level (*p* < 0.05, Fig. 2c). The results from Western blot analysis of control, T, 2-Hf, and T+2-Hf treated follicles confirmed major observations from the immunohistochemical analysis.

### Expression of mRNA for steroidogenic enzymes

**Fig. 3:**
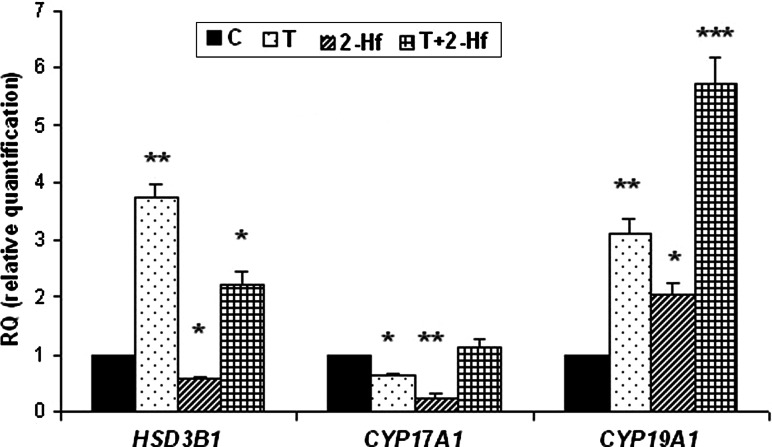
Relative expression of mRNA for 3β-HSD (HSD3B1) **a**, P450c17 (CYP17A1) **b** and P450arom (CYP19A1) **c** in control, testosterone (T), 2-hydroxyflutamide (2-Hf), testosterone and 2-hydroxyflutamide (T+2-Hf) treated whole follicles cultured for 24 h, using quantitative real-time PCR analysis. RQ (relative quantification) is expressed as mean ± SEM. *Asterisks* indicate statistically significant differences in the expression of steroidogenic enzyme genes between control and experimental groups (*p < 0.05, **p < 0.01, ***p < 0.001, Mann–Whitney’s test)

The expression of HSD3B1, CYP17A1 and CYP19A1 mRNA was detected in both control cultures and those stimulated with T, 2-Hf or T+2Hf (Fig. 3a–c). We utilized real-time RT-PCR analysis to quantitatively evaluate steroidogenic enzymes mRNA expression in follicular homogenates. Relative steroidogenic enzymes transcript levels in samples of experimental follicles were compared with the control, which was arbitrarily set at 1. As shown in Fig. 3 gene expression analysis revealed statistically significant differences in CYP17A1 mRNA levels, in follicles cultured with the addition of T and 2-Hf. In these samples the CYP17A1 transcript was statistically reduced, whereas no differences were found within groups treated with T+2-Hf. During follicular culture all applied factors caused statistical increase of CYP19A1 transcript. While, in case of HSD3B1, only T and T+2-Hf addition caused an elevated mRNA expression.

### Media steroid contents

**Fig. 4:**
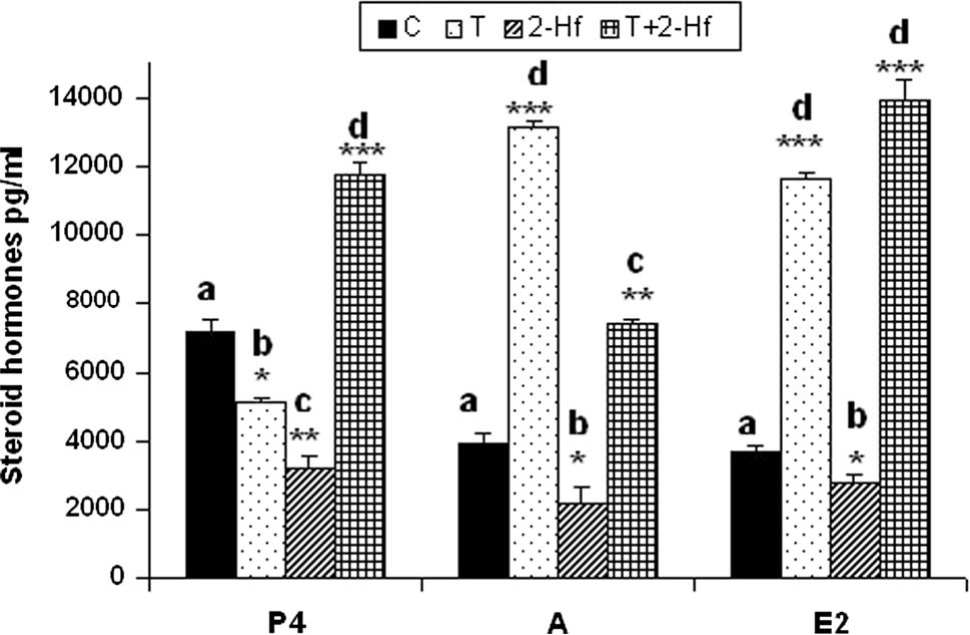
Progesterone, androgens and estradiol (mean ± SEM) secretion by whole follicles cultured for 24 h, respectively (*n* = 3 independent experiment). Statistical differences between control and experimental cultures were determined by Student *t* test. *Asterisks* indicate statistically significant differences (*p < 0.05, **p < 0.01). *C* control, *T* testosterone, *2-Hf* 2-hydroxyflutamide, *T+2-Hf* testosterone +2-hydroxyflutamide

Except for progesterone, addition of T caused a sharp increase in hormones level. Similarly, T+2-Hf media supplementation entailed an essential rise of progesterone, androgens and estradiol amounts. What is most interesting follicular 2-Hf treatment brought about dramatic decline in the production of these steroids (Fig. 4).

## Discussion

In the ovarian follicles of mammals, androgens are utilized as substrates for estrogen synthesis or can act via AR which can be detected immunohistochemically in granulosa and theca cells of preantral as well as growing antral porcine follicles. Within the ovary, granulosa cells generally display the strongest immunoreactivity for AR protein with the most intensive staining in the antral granulosa layer [30]. Also in culture AR immunostaining was detected in both, antral and mural granulosa cells isolated from porcine follicles at all stages of their development [7]. According to our prior results received thanks to the model system devised for studying agonism and antagonism of the AR [16], specific androgen receptor antagonist, 2-hydroxyflutamide caused AR overexpression but only in the presence of a very high level of androgen. The mechanism behind this finding remains unclear. 2-Hf interacted with the AR but this interaction seemed to be not cooperative in regulating the AR transcriptional machinery. It appears that 2-Hf has an ability to antagonize certain androgenic effects while increase others, which could be the effect of its interaction with AR. It is because 2-Hf- induced androgenic effects occur also in cytoplasmic signal transduction while conformational changes enable DNA binding and genomic response [31]. Based on this, the present study focused on the possible involvement of signaling through the AR on the induction and localization of key enzymes responsible for normal follicular steroidogenesis. Our results indicate beyond no doubt that, steroidogenic enzymes activity alike their expression is associated with the presence of androgens and AR in the porcine ovary.

Steroid hormones play an important role in the growth and differentiation of reproductive tissues as well as in the maintenance of pregnancy and fertility [4]. 3β-HSD, P450c17 and P450arom are important enzymes in the steroidogenic pathway leading to progesterone, androgens and estrogen synthesis, respectively [20, 21]. The results reported herein identified antiandrogen 2-Hf as an inhibitor of 3β-HSD and P450c17 protein and corresponding mRNA expression in cultured porcine follicles. What is more, after 2-Hf addition, their activity as measured by progesterone and androgens production was inhibited. Similar effect was observed in case of P450arom activity- estradiol secretion also dramatically declined. It seems possible, that AR blockade caused inhibition of steroidogenesis, which could be of clinical significance.

Androgen-induced follicular atresia is thought to occur by entry of androgens into the granulosa cells of ovarian follicles, where they bind to receptors and induce cell death [32, 33]. Androgens cause deterioration of follicles by increasing the number of pyknotic granulosa cells and degenerating oocytes. Antiandrogens prevent androgens from exerting their action on target tissues but it is not clear, whether they act only by blocking AR or also by inhibiting the biosynthesis of androgens. Some studies have suggested that FLU reduces androgen synthesis and/or increases its metabolism to inactive androgens [23, 34]. In our study the addition of 2-Hf resulted in significant reduction of levels of all steroids analyzed in the follicular media collected after culture. These findings support the hypothesis that 2-Hf directly blocks steroidogenic enzymes of all steroids synthesized in porcine follicles.

It is clear that 3β-HSD is the main enzyme involved in progesterone synthesis [35]. Our study shows that expression of HSD3B1 gene, which encodes 3β-HSD enzyme tended to increase with significant difference in porcine follicles cultured with the addition of T or T+2-Hf. It was also shown that 2-Hf caused an increase in HSD3B1 gene expression although without significant difference. Interestingly, in all experimental variants of follicular cultures, a dramatic decrease in 3β-HSD protein level was observed. Based on our previous data [33] this decrease was observed during progressive atretic stage of cultured follicle. Hormone measurement showed that the change in the pattern of progesterone level was mainly the same as 3β-HSD protein expression. Additionally, cytoplasmic PGR expression [36] increased significantly in progressively atretic follicles. These results suggest that there may be a functional coupling of 3β-HSD and AR expression and after antiandrogen treatment it is regulated to decrease progesterone synthesis directly leading to the progression of atresia.

The CYP17 gene encodes the cytochrome P450 17α-hydroxylase/c17,20-lyase enzyme, which uses pregnenolone or progesterone as a substrate for androgen synthesis [37]. Follicular androgens act as two way regulators during atresia. They directly accelerate atresia but they also allow follicles to maintain normal development, by acting as a substrate for estradiol synthesis [38]. Thus, a decrease in CYP17 during atresia may reduce estradiol production. Taken into consideration that the cytoplasmic localization of AR increases during atresia [16] this may improve androgen efficiency even after a decrease in CYP17 expression. Changes in CYP17 gene expression in porcine ovarian follicles after 2-Hf treatment were significant and showed tendency to decline. On the basis on our previous findings [33] we may state that CYP17 expression decrease is observed when follicles undergo progressive atresia. The main trend in CYP17 expression was consistent with the findings of Garret and Guthrie [39]. In rat testicular cells in vitro, FLU has been reported to inhibit 17,20-lyase activity of cytochrome P450 [23]. Activity of this enzyme has been detected in human thecal cells stimulated with LH and was high in polycystic ovaries [40]. FLU might reduce plasma androgen level by inhibiting activity of this enzyme.

The CYP19 encodes aromatase, which is an essential part of an enzyme system of vertebrate animals that is involved in the metabolism of androgens and ultimately their conversion to estrogen [41]. The synthesis of estrogens and the balance between androgens and estrogens level provided by aromatase plays a crucial role in the support of female reproductive differentiation and function. The pig remains unique among mammals investigated to date in expressing tissue- specific isoenzymes of P450 arom from at least three different genes [42]. It has been reported that Vinclozolin (Vnz) known as dicarboximide fungicide which has an antiandrogenic characteristics similar to that of FLU, was identified as an inducer of aromatase activity and mRNA expression in H295R human adrenocortical cells. Our data are consistent with this notion but only on mRNA level. Real time PCR analyses showed a marked increase in follicular P450arom mRNA expression after 2-Hf treatment with simultaneous loss of P450arom protein expression as well as its activity. It is possible that Vnz unlike 2-Hf may exert additional antiandrogenic properties via aromatase induction. Vnz is not known to interact with estrogen receptor or cause P450c17 inhibition, indicating that antagonism of AR is its main mechanism of function [43–45]. Consistent with the study by Huet et al. [46], CYP19 declined during atresia thus taken into consideration our previous studies [33] estradiol may play a major role in maintaining or promoting atresia.

In conclusion, we described the steroidogenic enzymes mRNA and protein expression profile in porcine medium follicles during an organ culture after exposure to an AR antagonist, 2-Hf. This was correlated with steroidogenic enzymes activity as indicated by steroid hormone production. We illustrated the associations among changes in these related factors and androgens, indicating androgens’ involvement in normal follicular function. The downregulation of key steroidogenic enzymes expression after exposure to 2-Hf, confirms its antiandrogenic and antiprogestational activity and demonstrates a key role for androgens in physiological granulosa cell apoptosis and porcine follicular atresia. These data seem useful for our current understanding of follicular atresia-specific factors and their interactions (at the level of medium-sized follicles) what in a consequence should provide a better insight into female fertility.
